
JOURNAL INFORMATION
==============================
NLM Title Abbreviation: Biospektrum (Heidelb)
Journal ID: Biospektrum (Heidelb)
NLM Journal ID: 9615685

Biospektrum

ISSN: 0947-0867
EISSN: 1868-6249

ARTICLE INFORMATION
==============================
PMCID: PMC7318718
PMID: 32834542
DOI: 10.1007/s12268-020-1408-0
Article ID: 1408
Article version: 1
Subjects: Karriere, Köpfe & Konzepte

Die Coronavirus Structural Task Force

Thorn Andrea andrea.thorn@web.de http://www.thorn-lab.de
1
1 grid.8379.5 0000 0001 1958 8658 Rudolf-Virchow-Zentrum - Center for Integrative and Translational Bioimaging, Universität Würzburg, Würzburg, Deutschland
2 grid.8379.5 0000 0001 1958 8658 Rudolf-Virchow-Zentrum, Universität Würzburg, Josef-Schneider-Straße 2, Haus D15, D-97080 Würzburg, Deutschland
Electronic publication date: 2020 Jun 26
Print publication date: 2020
Volume: 26
Issue: 4
First page: 442
Last page: 443
Copyright: © Die Autorin 2020
License: Open Access: Dieser Artikel wird unter der Creative Commons Namensnennung 4.0 International Lizenz veröffentlicht, welche die Nutzung, Vervielfältigung, Bearbeitung, Verbreitung und Wiedergabe in jeglichem Medium und Format erlaubt, sofern Sie den/die ursprünglichen Autor(en) und die Quelle ordnungsgemäß nennen, einen Link zur Creative Commons Lizenz beifügen und angeben, ob Änderungen vorgenommen wurden. Die in diesem Artikel enthaltenen Bilder und sonstiges Drittmaterial unterliegen ebenfalls der genannten Creative Commons Lizenz, sofern sich aus der Abbildungslegende nichts anderes ergibt. Sofern das betreffende Material nicht unter der genannten Creative Commons Lizenz steht und die betreffende Handlung nicht nach gesetzlichen Vorschriften erlaubt ist, ist für die oben aufgeführten Weiterverwendungen des Materials die Einwilligung des jeweiligen Rechteinhabers einzuholen. Weitere Details zur Lizenz entnehmen Sie bitte der Lizenzinformation auf http://creativecommons.org/licenses/by/4.0/deed.de.
License URL: https://creativecommons.org/licenses/by/4.0/

issue-copyright-statement: © Springer-Verlag GmbH Deutschland, ein Teil von Springer Nature 2020

==============================

■ Die moderne Biochemie ist ohne die genaue Kenntnis von Molekülstrukturen nicht vorstellbar: Protein-, RNA- und DNA-Strukturen ermöglichen uns Erkenntnisse über die chemischen Abläufe in lebenden Zellen. Über 160.000 bekannte Strukturen bilden den Schlüssel zu unserem Verständnis des Lebens.

Es gibt allerdings ein fundamentales Problem in der Strukturbiologie: Die dreidimensionalen Strukturen von Molekülen werden nicht direkt gemessen, sondern die Messdaten aus NMR (*nuclear magnetic resonance*), Kristallographie oder Kryo-Elektronenmikroskopie (Kryo-EM) müssen erst prozessiert und anschließend mit einem Strukturmodell interpretiert werden. Doch diese Modelle passen nie perfekt zu den Messdaten: Die Diskrepanz zwischen Modell und Messung wird z. B. in der Kristallographie als R-Wert in Prozent angegeben. Während Kleinmoleküle typischerweise Werte von fünf Prozent erreichen, sind es bei biologischen Makromolekülen 20–25 Prozent [1]. Diese verhältnismäßig schlechte Übereinstimmung macht es oft unmöglich, alle biologischen Fragen zu beantworten, und kann sogar die ganze Strukturlösung verfälschen. Das ist – zusammen mit der Komplexität von Strukturaufklärungen – der Grund, warum Fehler auch bei sorgfältiger Arbeit oft nicht zu vermeiden sind. Um Lösungen für diese Probleme zu finden, entwickelt meine Arbeitsgruppe an der Universität Würzburg neue Methoden: Wie können wir die Diskrepanz verringern und Strukturen besser modellieren? Wie können wir die Fehler in Messdaten, z. B. in der Kristallographie oder Kryo-EM, erkennen und reduzieren? Dank immer robusterer Methoden sind wir mittlerweile in der Lage, selbst schwierige, schlecht aufgelöste Strukturen großer Komplexe zu bestimmen.

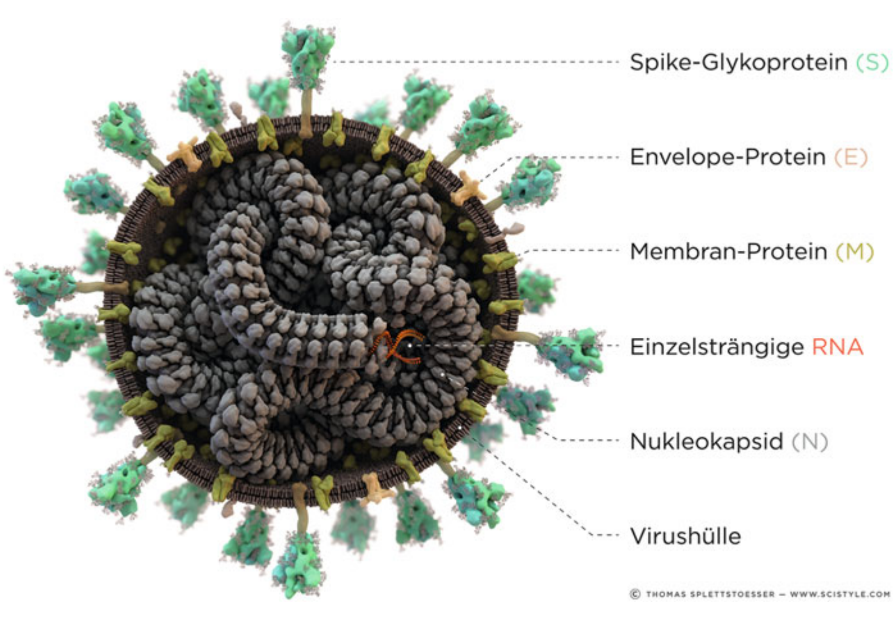

▲ **Abb. 1:** Abbildung des Coronavirus SARS-CoV-2 mit verschiedenen Proteinstrukturen. Nach Infektion der Wirtszelle bildet der Virus zusätzlich viele weitere Proteine, um sich zu replizieren. Bild: Thomas Splettstößer/SciStyle.com.

## Fehlerhafte Strukturen von SARS-CoV-2

Das Coronavirus SARS-CoV-2 hat die Welt gerade fest im Griff, und Proteinstrukturen spielen bei seiner Erforschung eine wichtige Rolle. Sie können Aufschluss über seinen Infektionszyklus geben und die gezielte Störung einzelner Coronavirus-Proteine durch strukturbasiertes Wirkstoffdesign ermöglichen. In den letzten Monaten hat die strukturbiologische Community schnell und umfassend reagiert und konnte viel zu unserem Wissen über das neue Coronavirus beitragen. Von 16 der 28 Proteine im Genom von SARS-CoV und SARS-CoV-2 gibt es mittlerweile (Teil-)Strukturen, insgesamt über 500 Strukturmodelle in der Proteindatenbank (PDB) [2]. Doch der hohe Zeitdruck fordert seinen Preis: Die Strukturen, die oft noch vor der Veröffentlichung in der PDB zur Verfügung gestellt werden, enthalten besonders viele Fehler.

Im Januar dieses Jahres habe ich zum ersten Mal makromolekulare Strukturen aus dem Coronavirus analysiert und festgestellt, dass diese mit heutigen Methoden wesentlich verbessert werden können. Zu diesem Zeitpunkt begannen viele Arbeitskreise weltweit gerade, nach Ansatzpunkten für die Entwicklung eines Wirkstoffes zu suchen. Ich brachte ein Team aus Methodenentwicklern und Studenten zusammen, um die Molekülstrukturen aus SARS-CoV und SARS-CoV-2 systematisch zu überprüfen und, wo möglich, zu verbessern. Wir fanden Probleme bei der Messung, wie Kontaminationen mit Eisstreuung, Strahlenschäden und fehlende Reflexe in kristallographischen Datensätzen, sowie Fehler in den Modellen, z. B. um bis zu neun Aminosäurereste verschobene Sequenzen in Kryo-EM-Strukturen, zu viele Ramachandran-Ausreißer in NMR-Strukturen, umgedrehte glykosidische Zucker und falsche Liganden. Jedes Mal, wenn wir eine Struktur verbessern können, stellen wir den Autoren das neue Strukturmodell zur Verfügung. Zudem sind die Daten auf unserer Seite insidecorona.net frei zugänglich, wo wir auch Hintergründe zur Funktion der Strukturen erklären.

## Daten für die internationale Forschung

Wir erhielten nach und nach Anfragen von Arzneimittelentwicklern überall auf der Welt. Unsere Daten werden als Grundlage für Molekulardynamikberechnungen von Folding@home [3] auf 1,4 Millionen Heimrechnern verwendet, in der EU-Initiative JEDI COVID-19 [4] zur Erforschung eines möglichen Arzneimittels, und sie sind in der Datenbank des MolSSI [5] integriert. Die *Coronavirus Structural Task Force* hat mittlerweile 18 Mitglieder aus sechs Ländern.

Jede Woche erscheinen neue Strukturen in der PDB. Unser Ziel ist es, dafür zu sorgen, dass sie so gut wie möglich sind. Dann stellen wir sie der internationalen Forschungsgemeinschaft zur Verfügung und leisten so unseren Beitrag zum Kampf gegen COVID-19. ■

Funding

Open access funding durch das Bundesministerium für Bildung und Forschung (Grant Number: 05K19WWA, Grant Acronym: AUSPEX).


Literatur

[1] Holton JM Classen S Frankel KA The R-factor gap in macromolecular crystallography: an untapped potential for insights on accurate structures FEBS J 2014 281 4046 4060 10.1111/febs.12922 25040949 PMC4282448
[2] Berman HM Westbrook J Feng Z The Protein Data Bank Nucleic Acids Res 2000 28 235 242 10.1093/nar/28.1.235 10592235 PMC102472
[3] Shirts M Pande VS Screen savers of the World Unite! Science 2000 290 1903 1904 10.1126/science.290.5498.1903 17742054
[4] Joint European Disruptive Initiative (JEDI), https://www.covid19.jedi.group
[5] The Molecular Sciences Software Institute (MolSSI), https://covid.molssi.org
